# Efficacy of Combined Ramosetron and Dexamethasone on Postoperative Recovery in Patients Undergoing General Anesthesia: A Multicenter Randomized Controlled Trial

**DOI:** 10.3390/jcm15135021

**Published:** 2026-06-27

**Authors:** Kuen Su Lee, Sang Hun Kim, Yoon Ji Choi, Eun-A Jang, Sun Yeul Lee, Jong Bum Choi, Jun-Mo Park, Hye Won Shin

**Affiliations:** 1 Department of Anesthesiology and Pain Medicine, Eulji University Uijeongbu Eulji Medical Center, Eulji University School of Medicine, Uijeongbu 11759, Republic of Korea; dlrmstnx@eulji.ac.kr; 2 Department of Anesthesiology and Pain Medicine, College of Medicine, Chosun University, Gwangju 61453, Republic of Korea; ksh3223@chosun.ac.kr; 3 Department of Anesthesiology and Pain Medicine, Korea University Ansan Hospital, Korea University College of Medicine, Ansan 15355, Republic of Korea; 4 Department of Anesthesiology and Pain Medicine, Chonnam National University Hospital, School of Dentistry, Chonnam National University, Gwangju 61469, Republic of Korea; sunsetglow@jnu.ac.kr; 5 Department of Anesthesiology and Pain Medicine, Chungnam National University Hospital, Daejeon 35015, Republic of Korea; neoquack@cnuh.co.kr; 6 Department of Anesthesiology and Pain Medicine, Ajou University School of Medicine, Suwon 16499, Republic of Korea; romeojb@aumc.ac.kr; 7 Department of Anesthesiology and Pain Medicine, Kyungpook National University Hospital, School of Medicine, Kyungpook National University, Daegu 41944, Republic of Korea; pjm4013@naver.com; 8 Department of Anesthesiology and Pain Medicine, Korea University Anam Hospital, Korea University College of Medicine, Seoul 02841, Republic of Korea; hwshin99@yahoo.com

**Keywords:** postoperative nausea and vomiting, ramosetron, dexamethasone, patient-controlled analgesia, multimodal prophylaxis, postoperative pain

## Abstract

**Background/Objectives:** Postoperative nausea and vomiting (PONV) is a frequent complication following general anesthesia. Ramosetron is a standard prophylactic agent for PONV; the efficacy of adjunctive dexamethasone in this specific population is not well established. We aimed to evaluate whether adding dexamethasone to ramosetron enhances antiemetic efficacy across diverse surgical procedures. **Methods:** This prospective, randomized, double-blind, multicenter trial enrolled adults undergoing gynecological, orthopedic, otolaryngologic, general, or plastic surgery managed without postoperative patient-controlled analgesia. We randomized 385 patients into two groups. Group D received 5 mg of dexamethasone immediately after anesthesia induction and ramosetron (0.3 mg) at the end of surgery, whereas Group C received only ramosetron. We assessed the incidence and severity of nausea and vomiting, pain scores, rescue antiemetic and analgesic requirements, and adverse events immediately after surgery and at 6 and 24 h postoperatively. **Results:** At 6 h, the incidence of nausea was significantly lower in Group D than in Group C (41.7% vs. 58.3%; *p* = 0.047). Group D also exhibited lower pain scores (VAS: 3.0 ± 1.8 vs. 3.5 ± 1.7; *p* = 0.012) and reduced consumption of additional analgesics (44.1% vs. 55.9%; *p* = 0.028). At 24 h, there were no significant differences between the two groups in the incidence of nausea, pain scores, or consumption of additional analgesics. Multivariable logistic regression analysis identified dexamethasone administration as an independent predictor of reduced postoperative nausea at 6 h (odds ratio 0.575; 95% confidence interval 0.344–0.962; *p* = 0.035). **Conclusions:** Low-dose dexamethasone to ramosetron substantially reduced postoperative nausea and improved analgesic profiles at 6 h in patients managed without PCA. However, no significant between-group differences were observed at 24 h.

## 1. Introduction

Postoperative nausea and vomiting (PONV) is a frequent complication in patients recovering from general anesthesia. PONV can lead to aspiration of gastric contents, increased intraocular pressure, patient discomfort, exacerbated pain, delayed recovery, prolonged hospitalization, and surgical site disruption, all of which contribute to poor prognoses [1,2].

The incidence of PONV is approximately 20–30% in the general surgical population but can escalate to 80% in high-risk groups [3,4]. Although the pathogenesis of PONV remains incompletely elucidated, its etiology involves patient-related, surgical, and anesthetic risk factors. Patient-related factors include young age, female sex, nonsmoking status, and a history of PONV or migraine. Surgical risk factors include laparoscopic, intra-abdominal, orthopedic, strabismus, breast, and plastic procedures. Anesthetic contributors include the use of volatile anesthetics, nitrous oxide, and opioids [5,6].

Given the multifactorial etiology of PONV, multimodal prophylaxis using agents with distinct mechanisms often provides superior therapeutic efficacy [7]. 5-Hydroxytryptamine type 3 (5-HT3) receptor antagonists are standard antiemetics recommended as first-line prophylactic therapy [8]. Ramosetron is a 5-HT3 receptor antagonist exhibiting higher receptor affinity and a longer duration of action than other class members [9]. Dexamethasone, a corticosteroid, is also a recognized antiemetic with demonstrated efficacy across various surgical procedures [10,11,12,13]. However, prolonged steroid use can induce adverse effects, including increased infection risk, hypertension, and hyperglycemia. Perioperative short-term dexamethasone administration is associated with elevated blood glucose levels. Furthermore, concerns persist regarding potential complications in high-risk patients [14]. Currently, dexamethasone has been widely used for PONV prophylaxis, with recommended doses ranging from 4 to 10 mg [15]. A previous meta-analysis categorized dexamethasone doses into low-dose (4–5 mg) and high-dose (8–10 mg) regimens [16]. Evaluating the ramosetron–dexamethasone combination has primarily been restricted to single-center trials or specific surgical procedures [17,18].

Furthermore, opioid consumption is one of the major risk factors for PONV. However, the amount of opioid administered via patient-controlled analgesia (PCA) is highly variable because it is influenced not only by patient-related factors such as age and comorbidities but also by non-patient-related factors such as patient dosing patterns, PCA settings, type of surgery, and the postoperative period, making it difficult to control for confounding [19,20,21]. Therefore, patients without PCA represent a population with relatively controlled opioid exposure.

Earlier PONV guidelines suggested that prophylaxis was not necessary for all patients, as low-risk patients were less likely to benefit and might be unnecessarily exposed to the potential adverse effects of antiemetics [7,22]. However, recent PONV guidelines encourage the use of combination antiemetic therapy even in patients with a relatively low predicted risk [15]. Because current PONV guidelines are designed for diverse surgical populations, we aimed to evaluate the effectiveness of the combination regimen in a pragmatic multicenter cohort representing a range of procedures associated with varying PONV risk.

Therefore, we designed a prospective, randomized, double-blind, multicenter study, hypothesizing that the combination of ramosetron and low-dose dexamethasone would reduce postoperative nausea compared with ramosetron alone in patients at high risk of PONV undergoing laparoscopic, gastrointestinal, orthopedic, strabismus, breast, or plastic surgery under general anesthesia without PCA.

## 2. Materials and Methods

We conducted this prospective, randomized, double-blind, multicenter study involving patients undergoing laparoscopic, gastrointestinal, orthopedic, strabismus, breast, or plastic surgery under general anesthesia at five university hospitals between March 2023 and August 2025. Participating sites included Korea University Ansan Hospital, Chonnam National University Hospital, Kyungpook National University Chilgok Hospital, Chungnam National University Hospital, and Ajou University Hospital. The study protocol was approved by the Institutional Review Boards of all participating institutions. We obtained written informed consent from all participants preoperatively. The trial was registered with the Clinical Research Information Service (registration number KCT0007007; https://cris.nih.go.kr/cris/index/index.do (accessed on 23 June 2026); registered on 18 February 2022) and conducted in accordance with the Declaration of Helsinki.

We enrolled adults (aged ≥ 19 years, American Society of Anesthesiologists physical status I–III) scheduled for elective laparoscopic, gastrointestinal, orthopedic, strabismus, breast, or plastic surgery under general anesthesia managed without PCA. Exclusion criteria included pregnancy, known hypersensitivity to the study drugs, hepatic or renal dysfunction, day care surgery and the use of antiemetics, steroids, antihistamines, or psychotropic medications affecting PONV within 24 h preoperatively. We also excluded patients with intraoperative complications requiring unplanned intensive care admission, which precluded protocol adherence.

We assigned participants in a 1:1 ratio to the combined group (Group D; ramosetron plus dexamethasone) or the control group (Group C; ramosetron only) using a computer-generated random allocation sequence. Randomization was performed using stratified block randomization according to surgical category (general surgery, gynecologic surgery, neuro-orthopedic surgery, plastic surgery, and otorhinolaryngology surgery) and participating hospital. The randomization list was generated and maintained by a researcher who was not involved in patient recruitment or outcome assessment.

An independent nurse prepared the study drug, dexamethasone, according to each group assignment. The study drug was administered as a single intravenous bolus of 5 mg of dexamethasone immediately after anesthesia induction. Dexamethasone has a delayed onset of action, and administration at the induction of anesthesia has been shown to provide greater protection against PONV than administration at the end of surgery [23,24]. All patients received 0.3 mg of intravenous ramosetron at the end of surgery. Blinded doctors who were not involved in the anesthesia assessment assessed clinical outcomes from the recovery room after surgery.

All participants underwent general anesthesia following a standardized protocol. We induced anesthesia with propofol (2 mg/kg) and maintained it with sevoflurane (2–3 vol%) in a 50% oxygen/air mixture. Rocuronium provided neuromuscular blockade, which was reversed using sugammadex or pyridostigmine combined with glycopyrrolate, as clinically indicated. Attending anesthesiologists administered remifentanil and fentanyl at their discretion.

The primary outcome was the incidence of postoperative nausea during the first 24 h postoperatively. PONV was assessed immediately after surgery and at 6 h and 24 h postoperatively by blinded assessors. PONV severity was evaluated using the Nausea and Vomiting Rating Scale (NVRS). Nausea was defined as a subjective urge to vomit and vomiting as the forceful oral expulsion of gastric contents. Patients requiring treatment for PONV received rescue antiemetics (intravenous metoclopramide, 10 mg) at the first episode. The severity of PONV was reassessed using the NVRS immediately before and 1 h after rescue administration. Secondary outcomes included pain intensity measured using the Visual Analog Scale (VAS), the number of patients requiring rescue antiemetic and additional analgesics immediately after surgery and at 6 h and 24 h postoperatively, as well as anesthesia time, surgical time and adverse events. Adverse events included shivering, headache, dizziness and drowsiness.

The minimum sample size was determined to be 190 based on an α-value of 0.05, a power of 0.80, calculated from the pilot data (incidence of nausea: R group: 46% and G group 33%) from 34 patients. Assuming a 10% loss to follow-up observations, a total of 400 patients were calculated to be required.

We compared categorical variables, including PONV incidence, using the Chi-square test or Fisher’s exact test. We analyzed non-normally distributed ordinal or continuous variables using the Wilcoxon rank-sum or Mann–Whitney U test. We conducted univariate and multivariable logistic regression analyses to identify independent predictors of PONV, adjusting for potential confounders. Variables with a *p* value < 0.15 in univariate analysis were included in the multivariable model. Statistical significance was set at *p* < 0.05. We performed analyses using the Statistical Package for the Social Sciences (SPSS), version 28 (IBM Corp., Armonk, NY, USA).

## 3. Results

**Figure 1:**
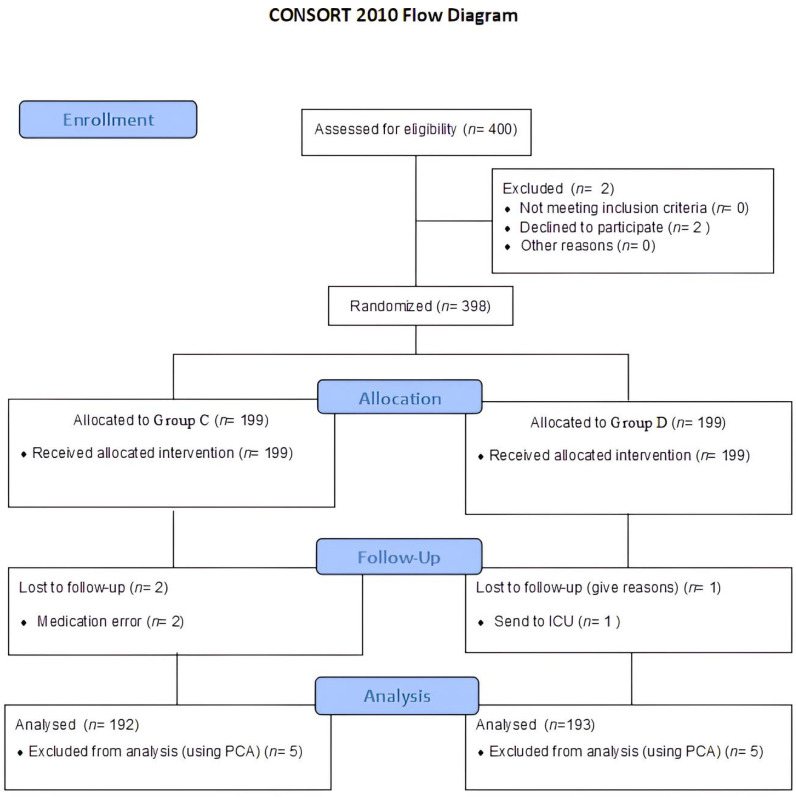
Consort flow diagram of this study.

Of the 400 patients who provided informed consent, two withdrew prior to randomization. We randomized the remaining 398 patients into Group D or Group C. Post-randomization exclusions included two patients in Group C (medication errors) and one in Group D (intensive care admission for massive bleeding). Additionally, ten patients (five per group) were excluded for protocol violations (receipt of PCA). Ultimately, the final analysis comprised 385 patients (Group C, *n* = 192; Group D, *n* = 193) (Figure 1).

Patient demographics, surgical types, and medical history (PONV, motion sickness, smoking status, and comorbidities) did not differ significantly between groups (Table 1). Anesthesia duration, laparoscopic use, and intraoperative analgesic consumption were comparable between groups.

Immediately postoperatively, we observed no significant intergroup differences in PONV incidence, nausea severity (NVRS), VAS scores, or rescue analgesic/antiemetic consumption. However, at 6 h, Group D exhibited a significantly lower incidence of nausea than Group C (41.7% vs. 58.3%; *p* = 0.047). Furthermore, Group D demonstrated significantly reduced VAS scores (3.0 ± 1.8 vs. 3.5 ± 1.7; *p* = 0.012) and analgesic requirements (44.1% vs. 55.9%; *p* = 0.028). At 24 h, outcomes were similar between groups except for analgesic consumption, which was significantly higher in Group D than in Group C (Group D, 54.4% vs. Group C, 45.6%; *p* = 0.033) (Table 2).

We performed logistic regression analysis to identify independent predictors of nausea at 6 h. Univariate analysis indicated that dexamethasone co-administration, sex, surgery type, anesthesia duration, intraoperative analgesic consumption, and immediate postoperative VAS scores correlated with PONV. Variables with a *p* value < 0.15 in univariate analysis were included in the multivariable model. In the multivariable model, dexamethasone co-administration (OR, 0.575; 95% CI, 0.344–0.962; *p* = 0.035), surgery type, and intraoperative analgesic consumption (OR, 27.513; 95% CI, 7.237–104.596; *p* < 0.001) remained independent predictors of PONV. Immediate postoperative VAS scores approached but did not reach statistical significance (*p* = 0.059) (Table 3).

## 4. Discussion

PONV is a prevalent complaint following surgery, often resulting in adverse physical and psychological sequelae. Patients often rate it as distressing as postoperative pain, significantly affecting the quality of recovery [25,26]. Consequently, effective PONV prophylaxis is crucial not only for patient satisfaction but also for postoperative prognosis. Given the multifactorial etiology of PONV, multimodal regimens targeting distinct mechanisms are physiologically superior to monotherapy. Combination therapy yields synergistic antiemetic effects [27,28,29,30]. Furthermore, our data confirms that combining ramosetron and dexamethasone does not increase the incidence of adverse effects in patients managed without PCA.

This study demonstrated that the incidence of nausea at 6 h postoperatively was statistically lower in the combination therapy group than in the ramosetron monotherapy group (*p* value = 0.035). However, there was no difference between the two groups in the incidence of nausea at 24 h postoperatively. Furthermore, the combined group exhibited lower VAS scores and reduced analgesic requirements than the control group at 6 h postoperatively. The combined group was shown to have a statistically lower analgesic requirement after 24 h, but there was no difference in VAS score.

The statistical difference between postoperative nausea and postoperative vomiting at 6 h postoperatively may be attributed to the following factors. Nausea occurs more frequently than vomiting, which may result in a more pronounced statistical difference. A meta-analysis reported that the prevalence of nausea ranged from 6.7% to 73.4%, whereas that of vomiting ranged from 2.2% to 37.5% [31]. In this study, due to the small number of patients who experienced vomiting, statistical significance may not have been achieved. The two symptoms are closely related clinically, and in this study, all patients who experienced vomiting also reported nausea. However, they have important pathophysiological differences. Nausea is a subjective sensation associated with conscious cortical activity, whereas vomiting is an autonomic reflex coordinated by the brainstem [32]. In a study by Stadler et al., risk factors for nausea and vomiting were found to overlap but were not identical; 11% of patients experienced nausea only, 2% experienced vomiting only, and 8% experienced both symptoms, suggesting that the two symptoms do not always occur together [6]. Meanwhile, some consider nausea to be a common prodromal symptom of vomiting.

Combining ramosetron and dexamethasone significantly reduces nausea incidence compared with ramosetron alone [17,27]. Although the precise mechanism of this interaction remains incompletely elucidated, proposed hypotheses include dexamethasone-mediated anti-inflammatory inhibition of serotonin release, depletion of the serotonin precursor tryptophan, and receptor sensitization, enhancing concurrent antiemetics [33,34,35]. However, the efficacy of this specific combination in diverse surgical populations managed without PCA had not previously been evaluated.

This combination reduced rescue antiemetic requirements more effectively than monotherapy in patients undergoing laparoscopic cholecystectomy in a previous study. [18]. However, that protocol utilized moderate-dose dexamethasone. Conversely, high-dose dexamethasone appears to prevent vomiting more effectively than low-dose regimens [36]. In contrast, no significant advantage of the combination over monotherapy was observed for PONV prevention in laparoscopic cholecystectomy, though rescue antiemetic consumption was significantly lower [37]. Notably, that analysis included only one study comparing the combination with ramosetron alone. Furthermore, no significant difference between the combination and monotherapy was observed in patients at low risk for PONV [38]. However, the limitation of that study to low-risk patients (≤2 risk factors) restricts its generalizability. Although our study design precludes a direct comparison between high- and low-dose regimens, this study showed a statistically lower incidence of postoperative nausea in the group receiving combined low-dose dexamethasone with ramosetron compared with ramosetron alone. Despite the short-term nature of this finding and the *p*-value being close to 0.05, the result supports further investigation, given the favorable safety profile of low-dose dexamethasone in high-risk patients.

In this study, when 0.3 mg of ramosetron was administered to patients undergoing diverse surgical procedures under general anesthesia with sevoflurane, the incidence of postoperative nausea was 33% immediately after surgery and 31% at 6 h postoperatively. This is slightly higher than the reported incidence of PONV, approximately 20–30%, in the general surgical population. This study included laparoscopic, gastrointestinal, orthopedic, strabismus, breast, and plastic surgeries, which are known to be associated with a high incidence of PONV [39,40,41,42]. Previous studies on prophylactic antiemetics have reported varying incidences of postoperative nausea depending on patient characteristics, surgical type, and anesthetic methods. Kim et al., in a multicenter study, reported the results of administering 0.3 mg of ramosetron to patients at low risk of developing PONV who underwent general anesthesia with sevoflurane [38]. In this study, the incidence of postoperative nausea was 9% at 0–1 h and 5% at 1–6 h postoperatively. In the study by Ryu et al., patients undergoing laparoscopic cholecystectomy under desflurane anesthesia received 0.3 mg of ramosetron, and the incidence of postoperative nausea was 36% at 0–2 h and 28% at 2–24 h postoperatively [18]. Another study reported that when 0.3 mg of ramosetron was administered to patients undergoing total knee arthroplasty under spinal anesthesia, the incidence of postoperative nausea was 37% at 0–6 h and 25% at 6–24 h postoperatively. These findings suggest that, despite prophylactic antiemetic use, the incidence of postoperative nausea may vary substantially according to patient characteristics and type of surgery. These findings suggest that, despite prophylactic antiemetic use, the incidence of postoperative nausea may vary substantially according to patient characteristics and the type of surgery. Therefore, although stratified randomization by surgical category was performed to minimize imbalance between treatment groups, residual clinical heterogeneity across different surgical procedures may have influenced the magnitude of the observed treatment effect. Nevertheless, the inclusion of a broad range of surgical procedures also enhances the external validity of our findings by reflecting routine clinical practice across multiple surgical specialties.

Glucocorticoids have demonstrated postoperative analgesic efficacy [43,44,45]. Compared with other analgesics, glucocorticoids exhibit a delayed onset, with effects typically emerging 3–6 h postoperatively [46,47,48]. This latency likely contributed to the reduced VAS scores observed in the combination group at 6 h in this study. Perioperative single-dose dexamethasone reportedly confers an opioid-sparing effect at moderate doses, but not at low doses [49]. However, in the present study, patients receiving low-dose combination therapy required fewer additional analgesics during the early postoperative period than those receiving monotherapy. These findings suggest a potential postoperative analgesic effect of low-dose combination therapy, although caution is warranted when interpreting the results. This is because the findings were transient and associated with a *p*-value close to 0.05. Although patients in the dexamethasone group required more additional analgesics at 24 h, pain scores at the same time point were comparable between the groups. These findings should be interpreted in conjunction with each other. Increased analgesic use may have contributed to maintaining pain scores comparable to those in the control group. In addition, patients in the control group required more analgesics during the early postoperative period, which may have reduced their subsequent analgesic requirements later during recovery. Because analgesic administration was determined according to clinical demand rather than a standardized protocol, analgesic consumption may not directly reflect pain intensity alone. The intraoperative opioid use was evaluated based on the number of patients rather than the amount administered. Because opioid exposure is a well-established determinant of both postoperative pain and PONV, variability in intraoperative opioid administration may have influenced the observed outcomes. Therefore, residual confounding related to intraoperative opioid dose cannot be completely excluded when interpreting the present findings.

Our study has limitations. First, we administered only low-dose dexamethasone, precluding comparison with high-dose regimens. Second, although stratified randomization according to surgical category was performed to reduce imbalance between groups, the inclusion of multiple surgical procedures inevitably introduced clinical heterogeneity. Therefore, residual confounding related to surgical characteristics cannot be completely excluded and may have influenced the observed treatment effects. Third, although the results of this study reached statistical significance, there may be concerns regarding whether the study was sufficiently powered to adequately account for potential correlations among risk factors. Fourth, this study did not include safety outcomes related to postoperative infection following dexamethasone administration. Previous studies suggest that dexamethasone administration is not significantly associated with an increased risk of postoperative infection [50]. However, additional consideration may be warranted in high-risk patients. Fifth, intraoperative opioid use was evaluated based on the number of patients who received opioids rather than the amount administered. This may have limited the accurate assessment of opioid effects and confounded both postoperative pain and postoperative nausea outcomes.

## 5. Conclusions

Combined low-dose dexamethasone and ramosetron resulted in a statistically lower incidence of postoperative nausea than ramosetron alone in patients undergoing surgery during the early postoperative period. Further research is needed to confirm the effect of combined low-dose dexamethasone and ramosetron on postoperative nausea.

## Figures and Tables

**Table 1:** Patients characteristics.

Patient Characteristics	Group C (*n* = 192)		Group D (*n* = 193)		*p* Value
Sex (Male/ Female), *n* (%)	66	(46.2)	77	(53.8)	0.262
Age (years)	49.2	±14.3	47.9	±15.1	0.403
BMI (kg/m^2^)	24.4	±3.7	24.7	±4.3	0.369
Surgery					0.934
General surgery, *n* (%)	40	(50.0)	40	(50.0)	
Obstetrics and gynecology surgery, *n* (%)	40	(50.0)	40	(50.0)	
Otorhinolaryngology surgery, *n* (%)	39	(50.0)	39	(50.0)	
Orthopedic surgery, *n* (%)	32	(45.7)	38	(54.3)	
Plastic surgery, *n* (%)	41	(53.2)	36	(46.8)	
Medical History, *n* (%)					0.492
History of PONV	3	(42.9)	4	(57.1)	
Motion sickness	22	(44.0)	28	(56.0)	
smoking	14	(58.3)	10	(41.7)	
Comorbidities					
Hypertension, *n* (%)	38	(46.3)	44	(53.7)	0.471
Diabetes mellitus, *n* (%)	17	(65.4)	9	(34.6)	0.101
Cardiac disorders, *n* (%)	3	(33.3)	6	(66.7)	0.315
Respiratory disorders, *n* (%)	6	(42.9)	8	(57.1)	0.593
Endocrine disorders, *n* (%)	6	(46.2)	7	(53.8)	0.785
Neurologic disorders, *n* (%)	8	(66.7)	4	(33.3)	0.237
Psychiatric disorders, *n* (%)	1	(25.0)	3	(75.0)	0.317
Renal/urinary disorders, *n* (%)	3	(50.0)	3	(50.0)	0.995

Data are presented as the number (%) of patients or mean ± standard deviation. BMI, body mass index; PONV, postoperative nausea and vomiting.

**Table 2:** Intraoperative and postoperative characteristics.

Characteristic	Group C (*n* = 192)		Group D (*n* = 193)		*p* Value
Anesthesia duration (min)	34.1	±44.4	40.0	±53.8	0.243
Laparoscopy, *n* (%)	21	(50.0)	21	(50.0)	0.986
Patients with intraoperative analgesic use, *n* (%)	119	(50.6)	116	(49.4)	0.706
Immediately after surgery					
Nausea, *n* (%)	63	(50.4)	62	(49.6)	0.885
Nausea severity (NVRS)	1.2	±1.9	1.1	±2.0	0.691
Vomiting, *n* (%)	3	(42.9)	4	(57.1)	0.708
VAS	4.1	±2.1	4.2	±2.2	0.654
Rescue antiemetic, *n* (%)	8	(34.8)	15	(65.2)	0.136
Additional analgesics, *n* (%)	50	(42.7)	67	(57.3)	0.064
6 h postoperatively					
Nausea, *n* (%)	60	(58.3)	43	(41.7)	0.047
Nausea severity (NVRS)	0.9	±1.6	0.8	±1.7	0.497
Vomiting, *n* (%)	6	(66.7)	3	(33.3)	0.308
VAS	3.5	±1.7	3.0	±1.8	0.012
Rescue antiemetic, *n* (%)	31	(55.4)	25	(44.6)	0.374
Additional analgesics, *n* (%)	99	(55.9)	78	(44.1)	0.028
24 h postoperatively					
Nausea, *n* (%)	36	(56.3)	28	(43.8)	0.264
NVRS	0.4	±1.0	0.3	±0.8	0.280
Vomiting, *n* (%)	1	(100.0)	0	(0.0)	0.315
VAS	2.1	±1.5	1.9	±1.5	0.107
Rescue antiemetic, *n* (%)	28	(59.6)	19	(40.4)	0.156
Additional analgesics, *n* (%)	108	(45.6)	129	(54.4)	0.033
Adverse events					
Shivering, *n* (%)	5	(71.4)	2	(28.6)	0.286
Headache, *n* (%)	4	(33.3)	8	(66.7)	
Dizziness, *n* (%)	2	(40.0)	3	(60.0)	
Drowsiness, *n* (%)	1	(100.0)	0	(0.0)	

Data are presented as the number (%) of patients or mean ± standard deviation. NVRS, nausea and vomiting rating scale; VAS, visual analog scale.

**Table 3:** Logistic regression analysis of factors associated with postoperative nausea at 6 h.

	Univariable				Multivariable			
		95% CI				95% CI		
	Odds Ratio	Low	High	*p* Value	Odds Ratio	Low	High	*p* Value
Group (ref:C Group)	0.616	0.390	0.971	0.037	0.575	0.344	0.962	0.035
Sex (ref:Female)	1.772	1.121	2.802	0.014	1.008	0.564	1.804	0.977
Age	0.989	0.974	1.004	0.158				
BMI (kg/m^2^)	1.014	0.959	1.072	0.623				
Surgery (ref:GS)								
OG	0.363	0.184	0.718	0.004	0.036	0.007	0.192	0.000
OL	3.723	2.210	6.272	0.000	0.214	0.028	1.664	0.141
OS	1.008	0.563	1.805	0.978	0.131	0.017	1.016	0.052
PS	0.936	0.530	1.651	0.818	0.359	0.054	2.398	0.290
Hypertension	0.773	0.437	1.369	0.378				
Diabetes mellitus	0.995	0.406	2.441	0.991				
Cardiac disorders	3.497	0.921	13.286	0.066				
Respiratory disorders	2.825	0.966	8.259	0.058				
Endocrine disorders	0.218	0.028	1.695	0.145				
Neurologic disorders	0.898	0.238	3.382	0.873				
Psychiatric disorders	2.735	0.380	19.674	0.318				
Renal/urinary disorders	2.752	0.547	13.859	0.220				
Anesthesia duration (min)	0.991	0.985	0.996	0.001	1.002	0.988	1.016	0.790
Laparoscopy	0.605	0.271	1.355	0.222				
Patients with intraoperative analgesic use, *n* (%)	4.233	2.419	7.406	0.000	27.513	7.237	104.596	0.000
Immediate postoperative VAS	1.181	1.061	1.315	0.002	1.128	0.996	1.279	0.059

Values are presented as odds ratios (ORs) with 95% confidence intervals (CIs). *p* < 0.15 for univariate-crude data. *p* < 0.05 for multivariate-crude data. BMI, body mass index; GS, general surgery; OG, obstetrics and gynecology surgery; OL, otorhinolaryngology surgery; OS, orthopedic surgery; PS, plastic surgery; VAS, visual analog scale.

## Data Availability

The raw data supporting the conclusions of this article will be made available by the authors on request.
